# Design of Fire Risk Estimation Method Based on Facility Data for Thermal Power Plants

**DOI:** 10.3390/s23218967

**Published:** 2023-11-04

**Authors:** Chai-Jong Song, Jea-Yun Park

**Affiliations:** Information Media Research Center, Korea Electronics Technology Institute, Seoul 03924, Republic of Korea; alboutyou@keti.re.kr

**Keywords:** fire risk estimation, SCADA system, anomaly detection, thermal power plant, turbine, boiler, exploratory data analysis

## Abstract

**Simple Summary:**

We provide a data classification and analysis method to estimate fire risk using facility data for thermal power plants. Experimental analysis is conducted on the data classified by the proposed method for 500 megawatt (MW) and 100 MW thermal power plants.

**Abstract:**

In this paper, we propose a data classification and analysis method to estimate fire risk using facility data of thermal power plants. To estimate fire risk based on facility data, we divided facilities into three states—Steady, Transient, and Anomaly—categorized by their purposes and operational conditions. This method is designed to satisfy three requirements of fire protection systems for thermal power plants. For example, areas with fire risk must be identified, and fire risks should be classified and integrated into existing systems. We classified thermal power plants into turbine, boiler, and indoor coal shed zones. Each zone was subdivided into small pieces of equipment. The turbine, generator, oil-related equipment, hydrogen (H2), and boiler feed pump (BFP) were selected for the turbine zone, while the pulverizer and ignition oil were chosen for the boiler zone. We selected fire-related tags from Supervisory Control and Data Acquisition (SCADA) data and acquired sample data during a specific period for two thermal power plants based on inspection of fire and explosion scenarios in thermal power plants over many years. We focused on crucial fire cases such as pool fires, 3D fires, and jet fires and organized three fire hazard levels for each zone. Experimental analysis was conducted with these data set by the proposed method for 500 MW and 100 MW thermal power plants. The data classification and analysis methods presented in this paper can provide indirect experience for data analysts who do not have domain knowledge about power plant fires and can also offer good inspiration for data analysts who need to understand power plant facilities.

## 1. Introduction

Several types of power plants, including nuclear, thermal, and hydroelectric, produce electricity. Any issues arising in these power plants can significantly impact the national economy and regional safety. Due to their crucial role in infrastructure, most nations consider power plants as essential facilities and prioritize their management accordingly. Power generation companies employ advanced systems to detect and resolve potential problems to ensure a continuous and safe electricity supply. Since fire risk is one of the severe problems in power plants, all power plant buildings have fire prevention systems to respond to fires. However, these systems employ a static method that assesses whether firefighting equipment, such as fire extinguishers and smoke–flame detectors, is present in a specific location, assigns each piece of equipment a particular score, and then calculates an overall score. Risk Failure Mode Effect Analysis (RFMEA) is a representative method that performs various types of risk analysis and evaluates identified risks based on severity, consequences, and likelihood of occurrence during the risk resolution process [1,2]. This risk assessment provides limited accuracy due to the rare occurrence of fires and diverse factors affecting fire occurrence. This traditional and static approach must be revised to detect and respond to fire hazards proactively. It is essential to create a fire protection system that can adapt to changes in fire risks over time. To achieve this, we can leverage the existing SCADA system in many power plants to incorporate time-based variables. This approach will enable dynamic detection and response to potential fire hazards. SCADA systems have become essential to the automated control and monitoring of critical infrastructure. They serve various purposes, such as monitoring facility status, acquiring large amounts of real-time data, increasing power efficiency, and automatically detecting facility abnormalities [3]. Due to their advantages, SCADA systems have become increasingly popular in facility abnormality detection research. Most studies using power plant SCADA data concentrate on power efficiency and predictive maintenance [4,5,6,7,8,9]. In order to develop a dynamic fire protection system, it is necessary to detect any abnormalities in the facilities. SCADA systems process complex time-series data, so analyzing multivariate time-series data is crucial to identify anomalies. In recent years, there has been rapid development in the research of multivariate time-series anomaly detection. Several systematic reviews have been conducted using deep-learning-based anomaly detection for multivariate time-series data. Anomalies in multivariate time-series can be defined in various ways, such as contextual anomalies, point anomalies, and interval anomalies, and there are numerous examples of industrial field applications and performance comparisons of various methodologies for detecting these defined anomalies [10,11,12,13]. Most of the turbine facilities that are crucial power plant components have rotating machines. As such, detecting abnormalities in these machines is essential, and we will explore this topic in this paper using SCADA data. A rotating machine’s two most critical components are the shaft and bearings. Bearings, in particular, play a crucial role in machines that rotate at high speeds, as damaged bearings can lead to equipment damage, explosions, or fires. Various deep learning methods are available to detect abnormalities, bearing defects, and the causes of vibration, and there are many examples of vibration measurement technology in the time, frequency, and time–frequency domains [14,15,16,17,18,19]. The boiler feed pump (BFP) and pulverizer are subject to fire risk prediction among the boiler equipment, and related research cases exist [16,20,21]. Although many studies have been conducted, most have focused on detecting facility abnormalities. Only a few have attempted to predict fire risk by identifying abnormal detection in power plant facilities [22]. Many of the studies introduced above are primarily studies of some facilities of thermal power plants or small-scale facilities such as wind power plants. For data analysis for each facility, it is essential to utilize the SCADA system for the entire thermal power plant. Additionally, in-depth data analysis is overlooked in most deep-learning-based research using SCADA data [23]. In order to effectively utilize a SCADA system, it is crucial to precisely analyze data from multivariate time-series and review various facility structures based on their function and location. This paper presents how to use SCADA data to develop a dynamic fire protection system that responds to a facility’s specific fire hazards based on location and function. The core of this paper is to identify fire-related data from SCADA data and utilize them through feature analysis of multivariate time-series data linked to the location and function of the facility. We analyzed and used two data sets from thermal power plants with 500 MW and 100 MW capacities for the experiments.

### 1.1. Contributions

From a fire prevention perspective, not all data from SCADA systems are necessary; only data related to fire risk are needed. However, mathematical and statistical methods still need to be available. The best way to do this is through collaboration between experts in fire risk and data analysts who have extensive experience with facility operation knowledge. This process takes a lot of time and effort and requires experts from various fields to work together. Also, even if deep learning technology is utilized, domain knowledge of power plant facilities and data analysis is necessary for better results. The data selection and analysis method described in this paper can help data analysts who lack domain knowledge of power plants better understand relevant time-series data. Additionally, it will inspire researchers to use SCADA data in various fields, not just predictive maintenance and anomaly detection, as SCADA data are utilized for fire risk estimation.

### 1.2. Structure of the Paper

The paper is organized as follows: Section 2 describes the design of the fire risk estimation method for a thermal power plant based on SCADA data. In Section 3, we present the classification of zones and facilities and the selection of tags used to analyze data for fire risk estimation. Section 4 outlines the experiments and discussions based on the classification and selection data. Finally, in Section 5, we provide the conclusions and future works.

## 2. Fire Risk Estimation Method for Thermal Power Plant

**Figure 1:**
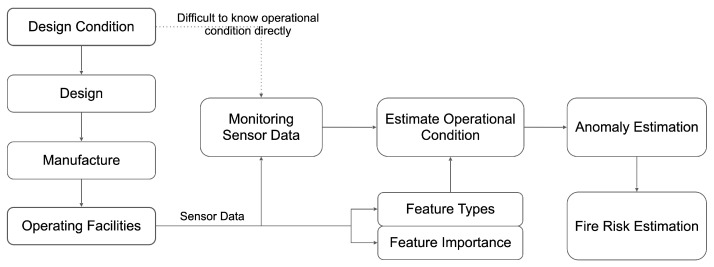
Approach for developing fire protection system.

**Figure 2:**
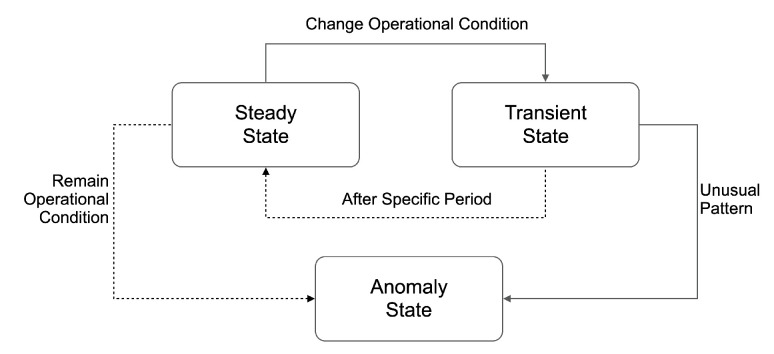
State modeling for facility data.

Our approach starts from the assumption that fire protection systems for thermal power plants can be developed using SCADA data, as described in Figure 1 [8,24]. All facilities in a thermal power plant are designed to achieve a specific purpose and operate under these conditions. There is no direct way to determine whether the facility operates under these conditions during operation. Therefore, the facility’s state must be indirectly monitored by installing sensors to ensure it works according to its requirements. Considering this perspective, Figure 2 can model the facility state. Sensor values stabilize within a specific range when a facility is operated under certain conditions, and the state remains the same. As operating conditions change, the corresponding sensor data also changes. Over time, the data eventually stabilize within a particular range of values. Repetition of this routine is the normal state of facility operation. However, if the sensor values change while the operating conditions remain the same or if they follow a pattern not seen in a normal state, this can be assumed to be an abnormal state [6,10]. Therefore, the state of the facility can be defined in three ways as follows:

- Steady State: This is when the operating condition remains constant, and the data are maintained within a specific range.
- Transient State: This is when the data change rapidly due to changing operating conditions. This change is expected.
- Anomaly State: This is when the operating conditions remain predefined, but the data change unexpectedly.

A comparison between transient and anomaly states is presented in Table 1.

### Fire Risk Estimation Method for Facilities

Generally, the causes of fires in thermal power plants can be classified into four categories: mechanical, electrical, chemical, and external reasons, such as carelessness during work. It is difficult to predict or prevent fires caused by carelessness at work through facility data analysis, and predicting fire risk from other factors is also challenging [2]. Although predicting fire risk directly from facility data is difficult, for fires caused by mechanical failure, it is possible to make some assumptions based on facility data.

- There is no fire risk if the facility is in normal condition.
- If the condition of the facility is abnormal, the probability of a fire occurring increases.
- There is no direct relationship between the facility’s abnormal condition and a fire’s occurrence. In other words, facility failure does not directly lead to a fire.

Therefore, estimating the fire risk of a facility requires a two-step approach: first, detecting anomalies in the facility and then exploring their relationship with fire.

## 3. Classification of Zones, Facilities, and Selection of Tags

### 3.1. Subdivision of Zone and Equipment

Because power plants have many facilities distributed over a large area, it is necessary to distinguish between areas prone to fire and those not. When designing a fire protection system based on facility data and additional environmental information, the following three considerations must be satisfied:

- Areas with a fire risk must be identified as much as possible to enable rapid response.
- Fire risks should be classified according to facility, workplace, and fuel type.
- Fire risk analysis should be integrated into existing and operational fire protection systems.

To meet these requirements, turbine and boiler areas containing critical equipment that can significantly impact plant facilities in the event of a fire were selected in consultation with plant officials. Turbines and boilers were classified as primary areas for fire risk prediction. An indoor coal shed area found only in coal-fired power plants was added. To subdivide the fire risk assessment for each facility in the turbine and boiler areas, the facility must be divided into smaller pieces of equipment. The types of equipment of the turbine and boiler zone were categorized by cooperating with fire experts based on analysis of power plant fire incidents over the years; it has been shown that much equipment in the turbine and boiler zones has been involved in severe fires. A turbine, a generator, oil, H2, and BFP were selected for the turbine zone, while the pulverizer and ignition oil were chosen for the boiler zone. The boiler system of a thermal power plant is very complex, consisting of various equipment such as burners, superheaters, reheaters, furnaces, air heaters, crushers, ignition oil, etc. Due to the nature of thermal power plants, much equipment used to handle steam and water poses a low and rare fire risk. The pulverizer is the equipment that causes most fires in the boiler zone. Additionally, with ignition oil, although the likelihood of a fire occurring is low, if a fire does occur, it can cause severe damage. So we selected only oil-related equipment and pulverizers and excluded the other equipment in the boiler zone. See Table 2.

We analyzed fire and explosion scenarios of thermal power plants over many years to classify the contents and ignition sources precisely and accurately. Fires in the turbine zone often were found in hydraulic oil and lubricant tanks, bearings, and oil supply pipes. These fires fall into three categories: pool fires, 3D fires, and jet fires. Extinguishing these fires can be challenging. At the same time, various types of fires were found in different equipment, including the pulverizer, lubricant oil, hydraulic oil tank, bearings, coal feeder, silos, feed piping, and air preheater in the boiler zone. Table 3 and Table 4 show ignition sources for fire scenarios in the turbine and boiler zones.

For coal-fired power plants, spontaneous combustion frequently occurs in coal supply devices and silos due to the nature of coal. However, these incidents have not been classified as severe fires. After carefully inspecting the turbine and boiler zones, we organized three fire hazard levels. Table 5 shows primary causes.

### 3.2. Tag Selection for Equipment

A 500 MW thermal power plant has more than 34,000 tags in the turbine and boiler areas, and a 100 MW has more than 20,000 tags. However, most of these tags are unrelated to fires, so selecting tags that may be related to fires is an essential process for more efficient estimation. No academic, mathematical, or statistical method can accurately identify fire-related data in SCADA data. Accordingly, we collaborated with field experts and collected opinions from facility operation experts to select appropriate tags in three stages. In the first stage, we excluded all tags not present in previous power plant fire cases, such as steam, air, various switches, and status indicators that detect conditions. This narrowed the selection to approximately 3000 and 1500 tags, respectively. In the second stage, tags related to minor fires that occurred in the past, such as water-related tags, tags related to abnormal internal temperatures of generators, and tags related to coal transportation at coal-fired power plants, were removed. Fewer than 1000 and 500 tags, respectively, were selected at this stage. Finally, based on the information summarized in Table 3, Table 4 and Table 5, we selected 183 tags in the turbine zone (Table 6) and 336 in the boiler zone for the 500 MW plant (Table 7) and 202 in the boiler zone for the 100 MW plant.

## 4. Experiments and Discussion

### 4.1. Data Set

The selected tags from the SCADA system were used to acquire data for a specific period corresponding to two power plants: one with a capacity of 500 MW and the other with 100 MW. Table 8 shows the data set description. The data contain 5%, 4%, and 3.5% missing values for each unit. Handling missing values in a data set is essential. Once power plant equipment begins operation, conditions typically remain the same until subsequent maintenance periods, which can last several months. As a result, sensor readings either do not change or follow the same pattern. Therefore, since the number of missing values in data sets is small, it is better to fill them with previous values rather than remove them. Using previously received values is also essential for algorithms that operate in real-time. The method of filling in missing values using the mean of the previous values of each sensor is as follows:

$$x_{n}=\frac{\sum_{k=1}^{N}x_{n-k}}{N}$$

Here, *x* represents the data series, *n* represents the current point, *k* represents the past point, and *N* represents the number of past points.

### 4.2. Main Features of the Facilities

The tags selected for fires in the turbine and boiler zone are mostly related to rotating machinery and oil. In the turbine zone, there are many tags for representative rotating machines such as HP, IP, LP, and generators [16]. The boiler zone contains the ignition oil and pulverizer. These facilities consist of rotating machines and have one thing in common: they use a lot of oil. Rotating machines comprise shafts, rotating bodies, bearings, and gearboxes, and various lubricants are used to ensure smooth rotation. Therefore, the essential characteristics of a rotating machine are the vibration and temperature of the shaft and bearings, the rotor’s position, and the lubricating oil’s temperature and pressure. Oil-related tags mainly include pressure, leakage, flow, and temperature.

Oil-Related Features

Lubricants and hydraulic oils are commonly used in turbines and boilers. However, due to their flammability, conducting a fire risk assessment to monitor these oils is essential. To this end, the SCADA system monitors the lubricant’s and hydraulic oil’s temperature and pressure at various points. These points include inside the oil tank, before passing through the cooler, and after being filtered for bearing lubrication. All the equipment tags of the turbine and boiler have oil-related features.

Bearing-Related Features

Bearings are essential for ensuring smooth machine rotation and come in various types. They must adequately operate rotating machinery by stably supporting the shaft and reducing friction during equipment rotation [14,15]. However, monitoring their state can be challenging because they are inside the structured form. Their vibration and temperature are indicators to determine the bearings’ state. Bearing vibration is measured using a contact accelerometer [17,25,26]. Tags for equipment such as HP, IP, LP, Generator, BFP, and Pulverizer include bearing temperature and vibration features.

Shaft-Related Features

Observing the vibration of a rotating shaft is crucial for accurately estimating the state of a rotating object. Perfect coupling is impossible because the shaft is fixed to the center of the rotating body through bearings. As a result, the rotating body vibrates in the x- and y-directions while displacing in the z-direction. A non-contact displacement sensor measures the vibration and position of the shaft, which are the most essential features of the shaft. Monitoring the shaft’s vibration and position during rotation is necessary, as even a stably rotating body has small vibrations [19]. Therefore, tolerance limits are established to differentiate between normal and abnormal vibrations. The operation is automatically stopped if the vibration exceeds the tolerance limit due to abnormal conditions. The HP, IP, LP, Generator, and BFP tags contain shaft vibration and position features.

Shell-Related Features

A turbine is a structure that consists of a rotating body fixed to a shaft and enclosed by a shell. When high-temperature steam is injected into the turbine, the shaft and shell expand in opposite directions based on the supporting points. However, if the degree of expansion of the shell and shaft is significantly different, it may cause the rotating body’s wings to collide with the shell, resulting in damage. Therefore, monitoring the shell expansion and the difference between the shell and shaft expansion is crucial. The HP, IP, LP, and Generator tags include shell expansion features.

H2-Related Features

A high-speed rotating turbine generates heat inside the generator, which requires a cooling system. Cooling systems for generators commonly use air, hydrogen, or water. Hydrogen is the preferred option for cooling in most power plants due to its numerous benefits.

- Due to its low density compared to air, H2 can flow easily in narrow gaps and experience minimal hydrodynamic loss.
- Mitigating facility deterioration through oxygen reduction helps prolong facilities’ lifespans.
- Its higher specific heat and heat transfer coefficient lead to a more significant cooling effect, resulting in higher output under the same conditions.

Although there are benefits to those above, there are also drawbacks.

- The concentration of hydrogen for cooling must be maintained above 97% due to its explosion range of 4~75% purity level.
- Installing additional facilities for leakage prevention and high-purity maintenance can prevent explosions but is costly.

Monitoring the H2 cooling system is essential for fire risk estimation due to its potential disadvantages. H2 tags include information on tank leakage, the collector, the cooler, purity, air temperature, pressure, and fan pressure difference.

### 4.3. Turbine Data

#### 4.3.1. Distribution and Correlation Analysis

**Figure 3:**
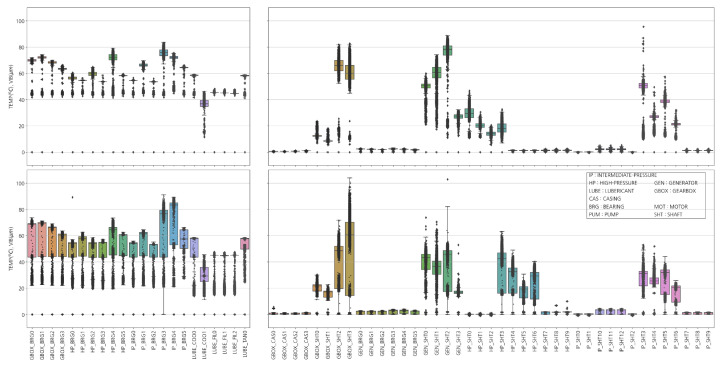
Distribution of turbine temperature and vibration: (**Upper Left**) temperature of Unit 1, (**Upper Right**) vibration of Unit 1, (**Lower Left**) temperature of Unit 2, and (**Lower Right**) vibration of Unit 2.

Figure 3 shows turbine temperature and vibration distribution data. The temperature data indicate that while the variation is slight in Unit 1, the overall variation is substantial in Unit 2. All states, except for variance, exhibit similar trends. The vibration data show identical patterns to the temperature data. The total variation in Unit 2 is more significant than that in Unit 1, which can be attributed to the unstable equipment condition of Unit 2, possibly due to its broader operating range or older age. However, IP_SHT3 and GEN_SHT2 in Unit 1 show a much wider vibration range than others. Furthermore, it is apparent that the vibration signals from HP_SHT0-6 are in different states from those of Unit 1 and Unit 2. This requires closer examination.

**Figure 4:**
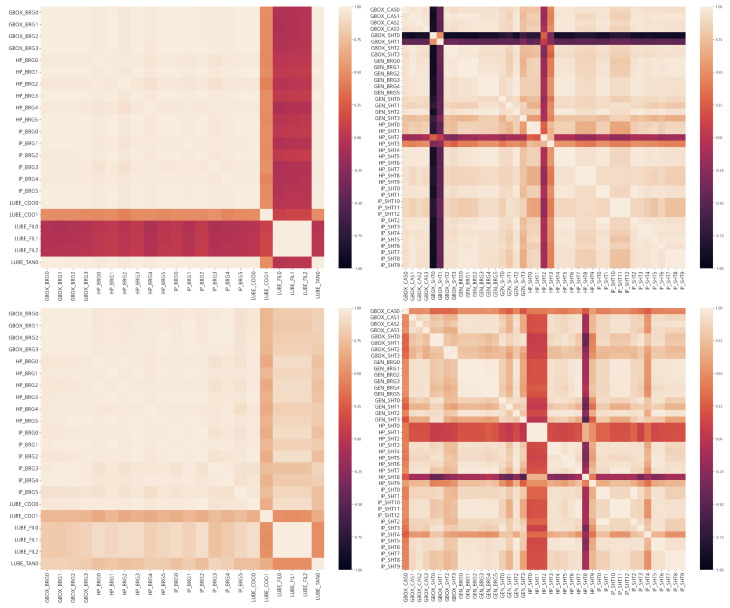
Correlation between turbines: (**Upper Left**) temperature of Unit 1, (**Upper Right**) vibration of Unit 1, (**Lower Left**) temperature of Unit 2, and (**Lower Right**) vibration for Unit 2.

Figure 4 shows the correlation between each sensor in the temperature and vibration data. The Pearson correlation coefficients calculated for each pair of columns are shown in Figure 4.

$$\begin{array}{cc} r_{xy} & =\frac{\sum_{i=1}^{n}\left(x_{i}-\bar{x}\right)\left(y_{i}-\bar{y}\right)}{\sqrt{\sum_{i=1}^{n}{\left(x_{i}-\bar{x}\right)}^{2}}\sqrt{\sum_{i=1}^{n}{\left(y_{i}-\bar{y}\right)}^{2}}}\\ \bar{x} & =\frac{1}{n}\sum_{i=1}^{n}x_{i}\\ \bar{y} & =\frac{1}{n}\sum_{i=1}^{n}y_{i} \end{array}$$

where *n* is the sample size, $x_{i},y_{i}$ are the individual sample points, and *i* is the index. The temperature patterns of Units 1 and 2 are similar, but the vibrations show significantly different patterns between the two units. In Unit 1, there is a strong correlation between the temperatures of the bearings. However, there is no direct relationship between the temperature of the lubricant and the temperature of the bearings. The temperature of the lubricant filter signal (LUBE_FIL), which is the temperature after passing through the cooler, is constant regardless of the turbine’s operating conditions. It is clear that the temperature of the lubricant positioned before the cooler shows significant correlation with the bearing temperature based on the operating conditions. In such cases, it would be beneficial to monitor the temperatures of the lubricating oil and bearings separately based on their locations. The relationship between the lubricant and bearing temperature in Unit 2 is more complex than in Unit 1. Specifically, the relationship is similar before and after the lubricant passes through the cooler. This suggests that the two facilities are operating in different operational conditions. The vibration data show a slightly different pattern compared to the temperature data. Specifically, only about four vibration sensors in Unit 1 operate independently, whereas in Unit 2, many sensors operate independently. This indicates that Units 1 and 2 might be functioning under different conditions.

#### 4.3.2. Characteristic Analysis

**Figure 5:**
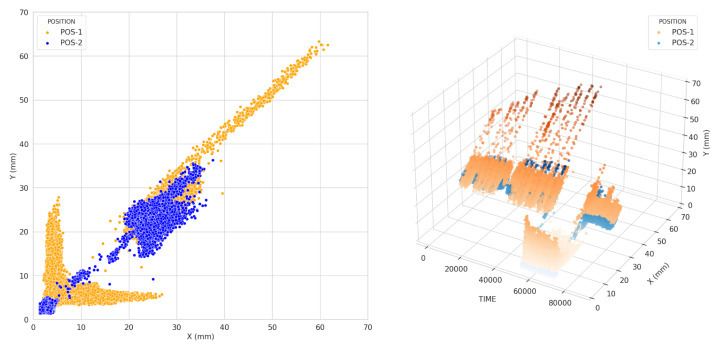
HP Shaft vibration of X- and Y-axes at Positions 1 and 2: (**Left**) scatter plot and (**Right**) 3D plot.

Figure 5 shows vibration data for shafts POS-1 and POS-2. The positions of POS-1 and POS-2 overlap, but POS-1 spreads in the X–Y-direction when the vibration is slight and gathers in one place when the vibration is significant. The vibration of POS-1 is distributed widely from 20 to 60 mm in the X- and Y-axes. On the other hand, the vibration of POS-2 is stably distributed below 40 mm. Observing and analyzing the details of the vibrations on a 3D rather than a 2D plot is easier.

**Figure 6:**
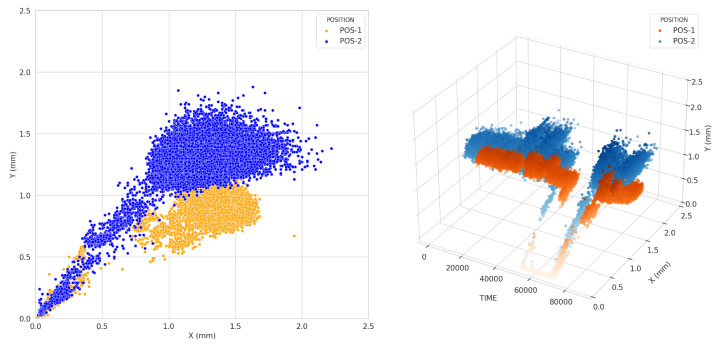
HP bearing vibration of X- and Y-axes at Positions 1 and 2: (**Left**) scatter plot and (**Right**) 3D plot.

Figure 6 depicts the different bearing vibration patterns compared to shaft vibration. Although there are similar vibration ranges on the X- and Y-axes for POS-1 and POS-2, POS-2 has a more extensive vibration distribution than POS-1 during normal turbine operation. Furthermore, more data than shaft vibration are observed, even when turbine operating conditions change during the transition phase. The bearing vibration condition varies relatively slowly compared to changes in operating conditions. The 3D plot also shows that the X-axis data are widely scattered, while the Y-axis data are concentrated in one place.

The remaining facilities, such as IP, LP, Generators, Lubricant Oil, H2, Hydraulic Oil, and BFP, were explored using the same procedure as HP.

##### Unusual Patterns

**Figure 7:**
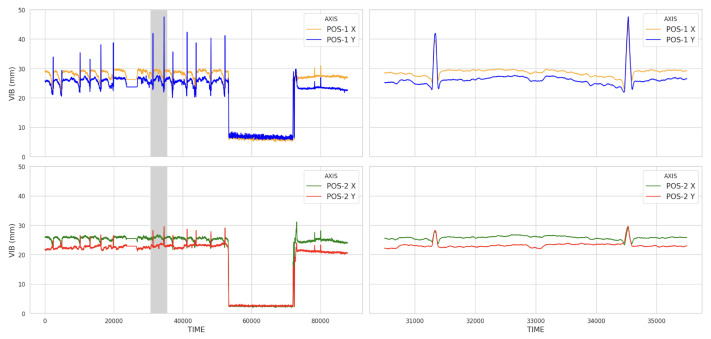
Unusual pattern of HP shaft vibration: (**Upper Left**) X- and Y-axes at Position 1, (**Upper Right**) zoom in on shaded areas of the left figure, (**Lower Left**) X- and Y-axes at Position 2, and (**Lower Right**) zoom in on shaded areas of the left figure.

The SCADA system is designed to automatically shutdown the turbine components when they exceed the preset alarm and trip values, which include vibration, temperature, position, and other factors [3,6]. However, as thermal power plants operate under stable conditions, it can be challenging for the SCADA system to detect abnormal patterns if sensor values do not exceed the preset thresholds [7]. Therefore, it is essential to carefully examine the data for any unusual patterns, even if the alarm and trip values are not reached. This helps to identify risk trends. Figure 7 shows the time-series data for HP turbine shaft vibration over a specific duration. We noticed a periodic increase in shaft vibration, which is an unusual pattern. This periodically increasing signal must be monitored, but no action is needed as it remains below the alarm threshold. It is believed that an action was taken during the overhaul period, as the signal disappeared afterward. As the area is enlarged to check the periodically occurring signal, it lasts about 2 h and then disappears. It is unclear why these signals occur periodically, but it is clear that they are unexpected.

### 4.4. Boiler Data

#### 4.4.1. Distribution and Correlation Analysis

**Figure 8:**
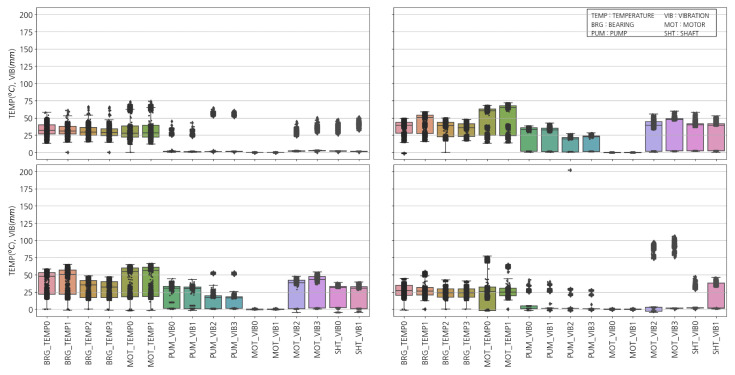
Distribution of BFP temperatures and vibrations: (**Upper Left**) Unit 1A, (**Upper Right**) Unit 1B, (**Lower Left**) Unit 2A, and (**Lower Right**) Unit 2B.

Figure 8 shows the BFP distribution data [20]. Units 1B and 2A have a more extensive range than Units 1A and 2B. It can be estimated that Units 1A and 2B do not undergo as many state transitions as Units 1B and 2A, and they are more stable. Unit 1A’s temperature distribution changes by 50% from 25 to 40 degrees. In Unit 2A, some sensors maintain their intermediate states for a considerable period. It can be confirmed that the MOT_TEMP0,1 and PUM_VIB2,3 sensors maintain a tri-state. This indicates that the BFP is operating at a medium level. In Unit 2B, the PUP_VIB3 vibrated over 200 mm, significantly higher than other sensors. Furthermore, MOT_VIB2,3 exhibited abnormally higher vibrations than all the other sensors. The operation of the BFP depends on the turbine’s condition and is not limited to the highest or minimum level.

- Different facilities show similar patterns under the same conditions but vary widely depending on their conditions.
- Two temperature states can be observed at identical locations, even if only a tri-state is observed by the vibration sensor.

**Figure 9:**
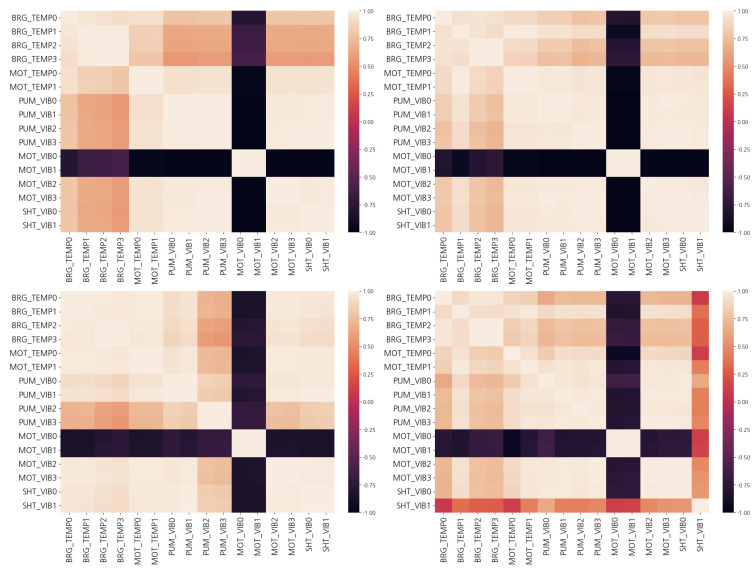
Correlation between BFPs: (**Upper Left**) Unit 1A, (**Upper Right**) Unit 1B, (**Lower Left**) Unit 2A, and (**Lower Right**) Unit 2B.

Figure 9 shows the correlation between temperature, vibration, and position of the BFP. It can be seen that a similar pattern is observed overall for each BFP. The correlation is relatively high for the same type of sensor in all facilities. It can be seen that the correlation between temperature and vibration is relatively high, while MOT_VIB0,1 has a high inverse relationship. Although the correlation between temperature sensors in Unit 1A is not observed as strongly as in Unit 1B, it can be confirmed that it is more than 70% overall. On the other hand, a more complex pattern is observed in Unit 2. A similar pattern is observed for each BFP. The correlation between the same type of sensor is relatively high in all units. Additionally, there is a relatively high correlation between temperature and vibration, while MOT_VIB0,1 shows a high inverse relationship. Although the correlation between temperature sensors in Unit 1A is not as strong as in Unit 1B, it can be confirmed that it is more than 70% overall. However, a more complex pattern is observed in Unit 2. Unit 2A’s overall correlation between vibration and temperature is robust. Unit 2B shows a slightly different pattern from Unit 2A. SHT_VIB0 correlates very highly with other sensors, while SHT_VIB1 does not correlate highly with other sensors. The remaining characteristics overall show similar correlation between Units 1A and B.

#### 4.4.2. Characteristic Analysis

As mentioned above, most of the equipment in the boiler zone was excepted except oil-related equipment and the pulverizer. Therefore, we focus on ignition oil and pulverizers among various equipment with high fire risk.

##### Ignition Oil

**Figure 10:**
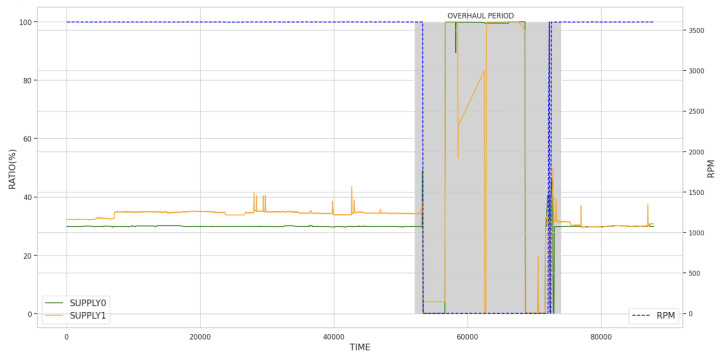
Supply path pressure values of ignition oil.

When the boiler starts, the ignition oil is only used once to ignite the burner. However, ignition oil has low density, which poses a high fire risk when it leaks, unlike lubrication or hydraulic oil with high viscosity. The data for ignition oil include supply and return path oil flow rates, temperatures, ratios, and pressures. During turbine operation, the oil temperatures in the supply and return paths show regular intervals. There is almost no temperature difference when the turbine stops. The supply path values are significantly different depending on the turbine operation conditions. However, the oil pressure fluctuates while the turbine is stopped, as shown in Figure 10. Operational experts acknowledged that the reason is related to equipment maintenance during the overhaul period. The sensor may detect unusual patterns when the facilities are dismantled and maintained. For these reasons, it is more reasonable to pause the monitoring of fire risk estimated from SCADA data during the overhaul period.

##### Pulverizer

**Figure 11:**
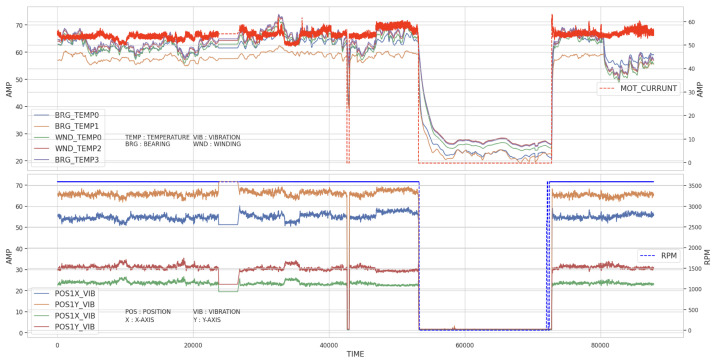
Pulverizer motor signals: (**Upper**) temperatures vs. current of motor and (**Lower**) vibrations of motor vs. turbine RPM.

The pulverizer grinds coal into fine particles, which are used to fuel the boiler burners in thermal power plants. However, the pulverizer is one of the facilities in the boiler zone that often causes fires. The system comprises various components, including the bunker, feeder, gearbox, motor, primary air, seal air, lubricant oil, hydraulic oil, and others, each monitored by numerous sensors. Due to the system’s complexity, nonlinearity, and high dimensionality, developing an accurate mathematical model for anomaly detection in the coal pulverizer system is challenging [21]. Therefore, pre-processing must be cautiously approached due to the large and diverse data. The bunkers, motors, lubricant oil, and gearboxes are the main objects that must be observed in the fire monitoring system. Grinding coal requires a lot of current caused by using a roller and motor. The pulverizer data strongly correlate with the motor’s current consumption, as shown in Figure 11. This is because the motor operation increases its load as current consumption rises regardless of the turbine’s state.

### 4.5. Discussion

After analyzing the data provided, we can gather the following information:

- The associated features are not altered if the RPM stays the same.
- Regarding the facility data for which we have not been provided with allowable ranges, the data of a single item cannot be meaningful because the permissible range is unknown.
- Observing the RPM can help us estimate how much the turbine-related features change. If there is a change to the RPM, we can predict the corresponding changes to turbine-related features. We then compare the expected and actual values. On the other hand, by observing the turbine-related features, we can estimate the current state of the turbine.
- Using RPM as a reference feature is appropriate due to its strong correlation with turbine-related features.
- Oil-related features exhibit diverse patterns and cannot be generalized. A deep learning model efficiently detects abnormalities.

The following items require additional discussion:

- What are the primary causes of data fluctuations compared to other units? Should these fluctuations be considered when designing the model if they fall within the normal range?
- Should different models be designed for each unit, even in identical facility types where different patterns may be observed?
- It is reasonable to assume that sensor data will have similar patterns. Therefore, is it appropriate to use one model to make predictions for the same facility?
- If abnormal sensor data are detected during the overhaul period, should the model automatically stop predicting for that period?
- When sensor data show an abnormal pattern, can a model distinguish between sensor malfunctions and equipment issues?
- How do we respond to facility abnormalities or deterioration based on the operating period, even under the same conditions?

**Figure 12:**
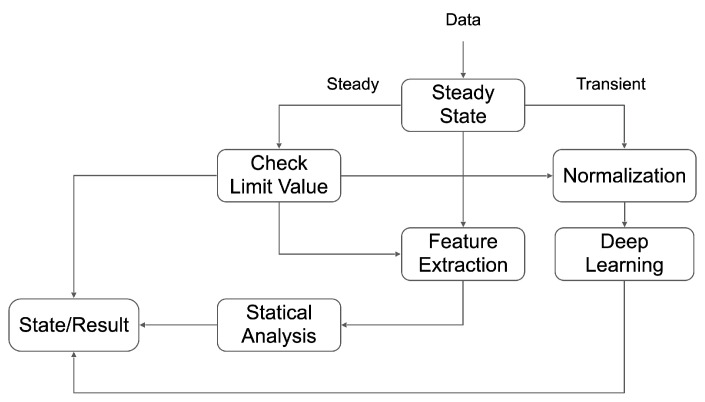
Fire risk estimation diagram.

We can design an algorithm for real-time fire risk estimation through the data analysis, as illustrated in Figure 12. The algorithm determines whether the equipment is being operated based on RPM sensor data. Statistical analysis can be conducted by extracting statistical features and checking the upper and lower limits when the system is operational. Along with statistical analysis, we also perform deep-learning-based analysis simultaneously. The deep-learning-based analysis is practical, but statistical analysis is used to explain anomalies. Fire risk is estimated by combining statistical analysis and deep-learning-based analysis.

## 5. Conclusions

In this paper, we proposed using facility data to estimate fire risk to develop a dynamic fire protection system for thermal power plants. Firstly, we considered that facilities are designed and manufactured with a specific purpose in mind and are operated within the scope of design conditions to achieve that purpose. Therefore, we presented a method for estimating fire risk using facility data. Since a facility’s operating conditions can determine its exact state, we assume it has normal, transient, and abnormal states and model fire risk estimation based on them. In order to ensure that the requirements for a dynamic fire protection system were met, the power plant’s facilities were categorized based on their functions, zones, and fuels. The corresponding data for each facility were classified accordingly. Furthermore, the selection of fire-related data from the SCADA data was explained to estimate the fire risk. In order to develop a fire risk estimation algorithm, the selected data were analyzed for their distribution, correlation, and characteristics of individual data for each facility. This helped us estimate the status and operation of the facility and understand the characteristics of the thermal power plant data. The data analysis was conducted using data sets from 500 MW and 100 MW power plants based on the described approach. The data classification and analysis method proposed in this paper can provide indirect experience to data analysts who need domain knowledge about power plant fires. It can also inspire data analysts who require knowledge of power plant facilities.

## 6. Future Works

In this paper, we proposed a method to select fire-related tags from the thermal power plant SCADA system and analyzed two plants’ data. Based on the analyzed results, research on machine learning and deep learning models that can estimate fire risk by facility and area should be conducted. Additionally, a framework that can store, process, and manage data in real-time based on classified tags should be developed. Since estimating fire risk based on facility data is very limited, better results can be produced when combined with image-based prediction, which is the most-actively researched method recently in deep learning.

## Figures and Tables

**Table 1:** Comparison between transient and anomaly states.

Transient State	Abnormal State	Remarks
Data changed	Data changed	By changing conditions or by remaining conditions
Repeated pattern	Unusual pattern	Change-point selection; change-pattern analysis
Changed in acceptable range	Changed in unacceptable range	Relative change according to operating conditions

**Table 2:** Subdivision of facility.

Zone	Facilities	Count
Turbine	High-Pressure (HP) Turbine, Intermediate-Pressure (IP) Turbine, Low-Pressure (LP) Turbine, Generator, H2, Hydraulic Oil, Lubricant Oil, BFP	8
Boiler	Ignition Oil, Pulverizer	2

**Table 3:** Fire and explosion scenarios and ignition sources in the turbine zone.

Equipment	Fire and Explosion Scenarios	Source of Ignition
Turbine Hydraulic Oil Tank	Pool fire due to oil leakage	High-temperature parts, Electrical cause, Hot work, Other
	Overheating due to low oil level in the tank	
	Oil overpressure	
	Oil overtemperature	
Turbine Lubricant Oil Tank	Pool fire due to oil leakage	High-temperature parts, Electrical cause, Hot work, Other
	Overheating due to low oil level in the tank	
	Oil overpressure	
	Oil overtemperature	
Turbine Bearing	3D fire caused by oil leakage	High-temperature parts, Electrical cause, Hot work, Other
	Oil oversupply	
	Oil overpressure	
	Oil overtemperature	
Generator Body	Unconfined Vapor Cloud Explosion (UVCE) due to H2 leakage	High-temperature parts, Electrical cause, Hot work, Other
	Jet fire due to H2 leakage	
	H2 overpressure	
	H2 oversupply	
H2 Supply Equipment	UVCE due to H2 leakage	
	Jet fire due to H2 leakage	
	H2 overpressure	
Lubricant and Hydraulic Oil Supply Piping	3D fire due to oil leakage	
	Pool fire due to oil leakage	
	Oil overpressure	
	Fire due to contact with high-temperature parts when oil is scattered	
Cable	Fire due to overheating cables	
	Other cable fires	
	Poor insulation due to water leakage	
Floor	Fire due to hot work	Welding, Cutting

**Table 4:** Fire and explosion scenarios and ignition sources in the boiler zone.

Equipment	Fire and Explosion Scenarios	Source of Ignition
Pulverizer Lubricant Oil Tank	Pool fire due to oil leakage	
	Oil level drop in tank	
	Oil overpressure	
Pulverizer Hydraulic Oil Tank	Pool fire due to oil leakage	
	Oil level drop in tank	
	Oil overpressure	
	Fire due to contact with high-temperature parts when oil is scattered	
Pulverizer Lubricant Oil Tank	Pool fire due to oil leakage	
	Oil level drop in tank	
	Oil overpressure	
Pulverizer Body	Pulverizer abnormal temperature	High-temperature part, Electrical cause hot work, Spontaneous ignition, etc.
	Sparks and fires in pulverizer	
	Dust explosion during initial start-up of the pulverizer	
	Spontaneous ignition during prolonged non-operation	
Coal Feeder	Abnormal temperature in the feeder	High-temperature part, Electrical cause hot work, Spontaneous ignition, etc.
	Spontaneous ignition in feeders	
	Fires in other feeders	
Silo	Abnormal temperature in silo	High-temperature parts, Electrical causes, Static electricity hot work, Spontaneous ignition, etc.
	Spontaneous fire in upper silo	
	Spontaneous fire of lower silo	
	Dust explosion by floating dust during coal loading	
	Other fires in silo	
Vacuum Refined Oil Supply Facility	Pool fire due to oil leakage	
	3D fire due to oil leakage	
Boiler Hydraulic Valve for Hydraulic Power Unit	Pool fire due to oil leakage	
	Fire due to contact with high-temperature parts when oil is scattered	
	Oil level drop in tank	
	Oil overpressure	
Lubricant and Hydraulic Oil Supply Piping	3D Fire due to oil leakage	
	Pool fire due to oil leakage	
	Oil overpressure	
	Fire due to contact with high-temperature parts when oil is scattered	
Air Preheater Reducer and Bearing	Pool fire due to oil leakage	
	Fire due to contact with high-temperature parts when oil is scattered	
	Anomaly caused by oil shortage	
Air Preheater Lubricant Oil Tank	Pool fire due to oil leakage	
	Overheating due to low oil level in the tank	
	Oil overpressure	
Boiler Ventilation Lubricator	Pool fire due to oil leakage	
	Fire due to contact with high-temperature parts when oil is scattered	
	Anomaly caused by oil shortage	
Floor	Fire caused by hot work	Welding, Cutting

**Table 5:** Primary causes of severe fires in thermal power plants.

Zone	1st Rank	2nd Rank	3rd Rank
Turbine	Lubricant Oil Leakage	Lubricant Oil Leakage	H2 Leakage
Boiler	Fuel Oil Leakage	Lubricant Oil Leakage	Lubricant Oil Leakage
Indoor Coal Storage	Spontaneous Combustion	Dust Explosion	General Fire

**Table 6:** Number of tags in turbine zone for 500 MW plant.

Zone	Equipment	Number of Tags	Features
Turbine	Turbine (Common)	9	Rotor position, number of revolutions, bearing oil pressure, hydraulic oil pressure
	HP	12	Shaft vibration and position, bearing temperature
	IP	12	Shaft vibration and position, bearing temperature
	LP-A~B	11 × 2	Shaft vibration and position, bearing temperature
	Generator	15	Shaft vibration, bearing temperature, air temperature
	H2	16	Cooler gas temperature, air temperature, H2 pressure, leakage rate
	Lubricant Oil	38	Bearing temperature, oil pressure, oil temperature
	Hydraulic Oil	7	Oil pressure, temperature, level
	BFP-A~B	30 × 2	Shaft position, oil and bearing temperature, speed

**Table 7:** Number of tags in boiler zone for 500 MW plant.

Zone	Equipment	Number of Tags	Features
Boiler	Ignition Oil	14	Pressure, flow, temperature
	Pulverizer (Common)	7	Pressure, valve position
	Pulverizer-A~F	53 × 6	Temperature, vibration, coal flow rate, number of revolutions, air flow rate, air pressure, hydraulic pressure, metal temperature, etc.

**Table 8:** Data set.

Generation Capacity	Zone	Number of Tags	Period	Interval
500 MW	Turbine	183	3 months	10 min
500 MW	Boiler	336	3 months	10 min
100 MW	Turbine	202	7 months	30 min

## Data Availability

Sample data sets are not available because of the NDAs of the thermal power plant companies.

## Abbreviations

The following abbreviations are used in this manuscript:

SCADA	Supervisory Control And Data Acquisition
AE	AutoEncoder
VAE	Variational AutoEncoder
LSTM	Long-Short Term Memory
OCSVM	One Class Support Vector Machine
IoT	Internet of Things
SVDD	Support Vector Data Description
SAD	Semi-Supervised Anomaly Detection
SVR	Support Vector Regression
AutoML	Automated Machine Learning
ECG	Electrocardiogram
MW	Megawatt
HP	High-Pressure
IP	Intermediate-Pressure
LP	Low-Pressure
BFP	Boiler Feed Pump
H2	Hydrogen
RPM	Revolutions Per Minute
UVCE	Unconfined Vapor Cloud Explosion
BOP	Balance of Plant
